# Manual of Hypodermic Medication

**Published:** 1891-08

**Authors:** 


Manual of Hypodermic Medication. By Drs. Bourneville and Bricou. Translated by G. Archie Stockwell, M. D., F. Z. S., member of the New Sydenham Society, London. 1890. George H. Davis, Detroit, Mich. Pp. 158. Price, in paper, 25 cents; in cloth, 50 cents. The Physicians' Leisure Library.

The original work by the well-known French authors achieved such success in France that it was deemed advisable to translate it, so that English-reading physicians could scan its pages. The formulæ of the various medicaments are written according to the English system only, whereas, had both been given, it might have been more desirable. A fact perhaps not very generally known is where absorption takes place most rapidly. According to Lampert, Eulenburg, and Davis, absorption occurs most rapidly when introduced in the temples or cheeks; next, in the epigastrium; third, in the anterior portion of the thorax; fourth, inner side of arm and thigh; fifth, nape, etc.

It makes a convenient little work for reference.

